# Improving person-centred care in nursing homes through dementia-care mapping: design of a cluster-randomised controlled trial

**DOI:** 10.1186/1471-2318-12-1

**Published:** 2012-01-03

**Authors:** Geertje van de Ven, Irena Draskovic, Eddy MM Adang, Rogier ART Donders, Aukje Post, Sytse U Zuidema, Raymond TCM Koopmans, Myrra JFJ Vernooij-Dassen

**Affiliations:** 1 Department of Primary and Community Care, Radboud University Nijmegen Medical Centre, P.O. Box 9101, 117 ELG, 6500 HB Nijmegen, The Netherlands; 2 Department of Epidemiology, Biostatistics and HTA, Radboud University Nijmegen Medical Centre, P.O. Box 9101, 113 EBH, 6500 HB Nijmegen, The Netherlands; 3 Dementia-care mapping, The Netherlands, De Friese Wouden, P.O. Box 215, 9250 AE Burgum, The Netherlands; 4 Scientific Institute for Quality of Healthcare, Radboud University Nijmegen Medical Centre, P.O. Box 9101, 114 IQ healthcare, 6500 HB Nijmegen, The Netherlands; 5 Kalorama Foundation, Postbus 85, 6573 ZH Beek-Ubbergen, The Netherlands

## Abstract

**Background:**

The effectiveness and efficiency of nursing-home dementia care are suboptimal: there are high rates of neuropsychiatric symptoms among the residents and work-related stress among the staff. Dementia-care mapping is a person-centred care method that may alleviate both the resident and the staff problems. The main objective of this study is to evaluate the effectiveness and cost-effectiveness of dementia-care mapping in nursing-home dementia care.

**Methods/Design:**

The study is a cluster-randomised controlled trial, with nursing homes grouped in clusters. Studywise minimisation is the allocation method. Nursing homes in the intervention group will receive a dementia-care-mapping intervention, while the control group will receive usual care. The primary outcome measure is resident agitation, to be assessed with the Cohen-Mansfield Agitation Inventory. The secondary outcomes are resident neuropsychiatric symptoms, assessed with the Neuropsychiatric Inventory - Nursing Homes and quality of life, assessed with Qualidem and the EQ-5D. The staff outcomes are stress reactions, job satisfaction and job-stress-related absenteeism, and staff turnover rate, assessed with the Questionnaire about Experience and Assessment of Work, the General Health Questionnaire-12, and the Maastricht Job Satisfaction Scale for Health Care, respectively. We will collect the data from the questionnaires and electronic registration systems. We will employ linear mixed-effect models and cost-effectiveness analyses to evaluate the outcomes. We will use structural equation modelling in the secondary analysis to evaluate the plausibility of a theoretical model regarding the effectiveness of the dementia-care mapping intervention. We will set up process analyses, including focus groups with staff, to determine the relevant facilitators of and barriers to implementing dementia-care mapping broadly.

**Discussion:**

A novelty of dementia-care mapping is that it offers an integral person-centred approach to dementia care in nursing homes. The major strengths of the study design are the large sample size, the cluster-randomisation, and the one-year follow-up. The generalisability of the implementation strategies may be questionable because the motivation for person-centred care in both the intervention and control nursing homes is above average. The results of this study may be useful in improving the quality of care and are relevant for policymakers.

**Trial registration:**

The trial is registered in the Netherlands National Trial Register: NTR2314.

## Background

The prevalence of neuropsychiatric symptoms among nursing-home residents with dementia is about 80% [1-4]. In addition to directly affecting the residents' quality of life, these symptoms represent a serious challenge to professional caregivers [5,6]. Staff job dissatisfaction results in high illness absenteeism (5.4%) and turnover rates, which ultimately leads to staff shortages [7-13]. A strong relationship has been found between high staff turnover and poor resident outcomes such as quality-of-care deficiencies, quality-of-life deficiencies, use of psychoactive drugs, and drug-induced hospital admission due to serious adverse events [6,8,14,15]. These facts suggest that the current efforts put into dementia care leave room for improvement in quality and cost-effectiveness of care. In order to provide optimal dementia care, the staff often needs additional training [13,16-18]. Dementia-care mapping (DCM) is a multicomponent intervention, which was developed by the Dementia Research Group at Bradford University, UK, in 1992, and is based on Kitwood's social-psychological theory of personhood in dementia [19]. This theory posits that much of the ill-being that people with dementia experience is due to negative environmental influences, including staff attitudes and care practices. Dementia-care mapping assists staff in identifying the triggers causing the well-being and ill-being of people with dementia [20].

Dementia-care mapping offers an integral, person-centred approach to dementia care. Many other interventions based on person-centred care, such as multimodal sensory stimulation (snoezelen) [14,21] and person-centred bathing [22,23] have a more limited scope. These interventions aim either at residents or at staff alone, and while they are very valuable in their own right, they are limited to psychosocial aspects of care or they apply in a single care-giving situation such as bathing. These interventions often do not include systematic adaptations in management style and organisational climate. We can expect single-scope interventions, usually aimed either at staff, residents, management style, or organisational climate alone, need to operate synergistically if we are to sustainably improve effectiveness, efficiency, and quality of dementia care in nursing homes. Dementia care experts recommend using a range of interventions that address the needs of both residents and staff [24]. The aims of this study are to reduce the frequency and intensity of neuropsychiatric symptoms, improve the quality of life of dementia patients, improve staff-resident interactions and staff job satisfaction, and reduce job-related stress by means of the introduction of the DCM method in dementia care. We will use a cost-effectiveness analysis to determine whether the intervention positively affects the efficiency of care.

## Methods/Design

### Study design and setting

**Figure 1:**
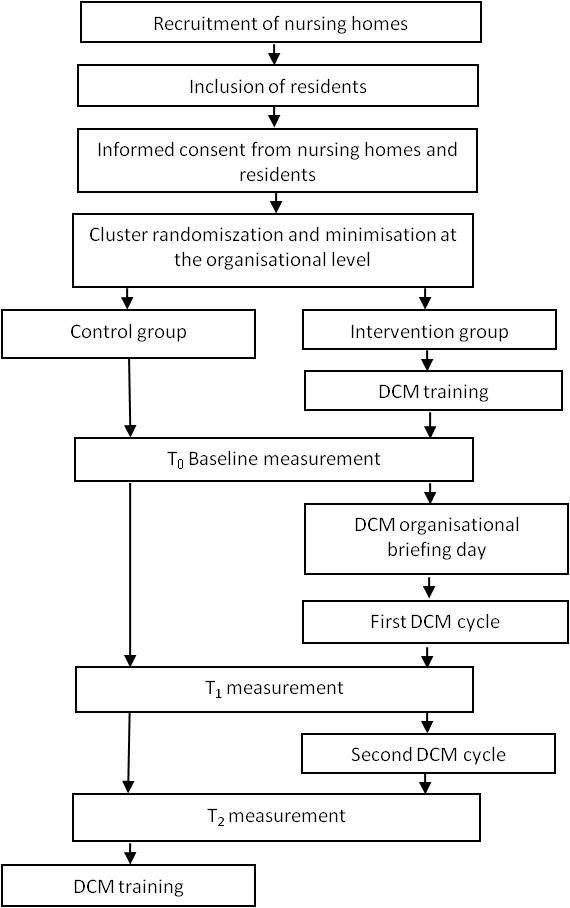
**Study design**.

The study is a cluster-randomised, controlled trial (Figure 1). We will evaluate the DCM intervention in Dutch nursing homes, which will be clustered. We will use cluster-randomisation in order to avoid contamination with the effects of possible exchange of information within a cluster. We will use a studywise minimisation method [25] to allocate the clusters (units) to either the intervention group or the control group. Nursing homes in the intervention group will receive DCM training and a DCM organisational briefing day. Care will be evaluated in two DCM cycles of observation, feedback, and action plans. Quantitative methods will be used to study effectiveness and efficiency, and qualitative methods will be used to conduct a process analysis and to study facilitators of and barriers to broader implementation of DCM in daily practice. The ethical committee Arnhem-Nijmegen waived approval for this study (registration number 2010/147).

### Study sample

The study sample will consist of residents with dementia from nursing-home dementia special-care units (DSCUs) and their formal caregivers. Now, at the time of writing, the nursing homes have been recruited. This was done in several ways: e.g. advertising on the Dutch DCM website http://dcmnederland.nl/, the VENVN website (the website of a Dutch professional organisation for nursing personnel), and invitational letters to nursing homes with information about the project.

We recruited 34 DSCUs from 11 nursing home organisations. The participating nursing homes serve several regions in the Netherlands. A DSCU is defined as a residential unit with common areas and staff. This can be a group in a small-group residential facility or a DSCU in a nursing home. The number of patients in a DSCU can range from 3 to 32. The participating DSCUs will provide residence for at least 250 people. The inclusion criteria for the residents are as follows:

• Age of 65 years or more

• Dementia diagnosed by an elderly-care physician according to the *Diagnostic and statistical manual of mental disorders-IV *criteria for dementia [26]

• Approval of the elderly-care physician for inclusion

• At least one of the following neuropsychiatric symptoms: aggression, motor or verbal agitation, psychosis, depression, and apathy

• Informed consent given by the residents themselves, their families, or their legal guardians

• The resident must use the common areas, such as the shared living room, at least 4 hours a day.

Residents with an estimated life expectancy of 6 weeks, or those who are physically unable to spend time in common areas of the facility, will not be included in the study. If residents withdraw their consent for any reason or develop a life-threatening disease, they will be excluded from the study. Evidence shows that the attrition rate is relatively high in this kind of population, so, to allow for intention-to-treat analysis, we will replace any participants lost to follow-up with new participants.

### Bias control and randomisation

Randomisation will take place after the study sample has been recruited and informed consent has been given, but before the DCM training, the DCM organisational briefing day, and the start of the intervention. The clusters will be randomised to avoid contamination by the effects of possible exchange of information within a nursing home. The dementia care mappers will be recruited from DSCUs other than those where the DCM cycles will take place. The reason for this is that the DCM observations and feedback should not be influenced by professional or personal relationships. The minimisation method will be used for randomisation [25] to assure an equal distribution of baseline characteristics to the intervention and control groups. This means that nursing homes will be randomised with the aid of adaptive weights based on the sizes of the nursing homes, DSCU sizes and the formal caregiver-to-resident ratios. Nursing homes will be randomly allocated to one of two conditions: the DCM intervention and usual care. A person who has no knowledge of and no relationship to the study will do the randomisation with appropriate software to assure allocation concealment.

Because of the DCM training and intervention, the study cannot be blinded with respect to nursing homes, residents, and their caregivers. The researcher (GV), the research assistant (FB), and the DCM trainer (AP) will not be blinded to this information.

### Intervention

The Bradford Dementia Group [27] developed the DCM method, which is based on the principles of person-centred care [28,29]. The DCM method is an observational tool that has been used in formal dementia care settings since 1992, both as an instrument for developing person-centred care practice, and as a tool in evaluative research [20,30,31]. Dementia-care mapping is a method in which care improvement plans (action plans) are based on systematic observations of the actual care as it takes place in formal settings such as nursing homes and day care. The feedback to the staff is expected to raise their awareness regarding the interdependency of their own behaviour and that of the residents. The feedback occurs in a nonthreatening way and does not serve as staff-evaluation tool. The fact that not only 'negative' but also 'positive' events are recorded and brought to light motivates staff to improve their competences and performance. Dementia-care mapping offers a set of tools for personal and organisational development. Through DCM, the staff may attain an important signalling role towards the members of the multidisciplinary care teams in nursing homes (which include psychologists, elderly-care physicians, regular physicians, physiotherapists, and occupational therapists). This allows for the timely initiation of tailor-made psychological or other interventions [32], which is very important in ensuring long-term positive effects of DCM. Furthermore, it is important to emphasise that the DCM method acts as a channel for the timely implementation of various kinds of improvements for individuals (residents and caregivers) groups (professional development needs), DSCUs, multidisciplinary teams, management, and organisations. This way, the improvement actions become well coordinated and sufficiently individually tailored.

### Intervention components of dementia-care mapping

#### Phase 1: training in dementia-care mapping

Staff members of intervention nursing homes will receive DCM training. A basic DCM user needs a 4-day course of basic concepts and skills. A basic user can participate in a DCM team under the supervision of an advanced user. To become an advanced user, a staff member must also take a 3-day course about the background and theory of DCM. Advanced users can map care, report observations, lead a DCM team, give feedback to the staff, and instruct and support them in drawing up action plans. At least one staff member in each organisation will become an advanced user.

#### Phase 2: organisational briefing day for dementia-care mapping

At the end of the DCM training, intervention nursing homes will be visited and will receive a one-day training course. This course provides organisation-wide basic understanding of the DCM method to ensure endorsement of DCM goals and methods and to aid its implementation in an organisation or setting.

#### Phase 3: two dementia-care mapping cycles: observations-feedback-action plan

**Figure 2:**
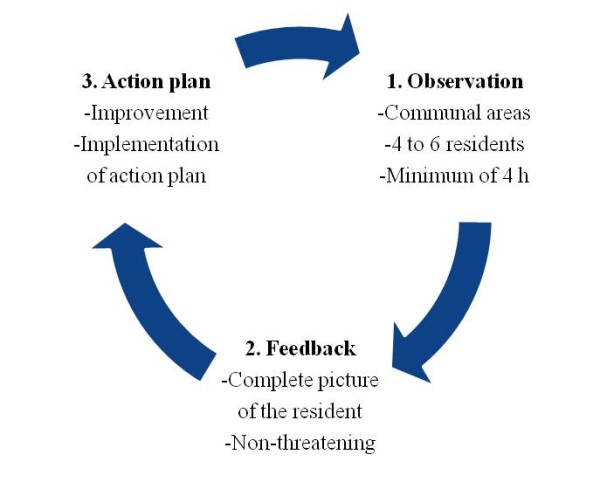
**Single cycle of dementia-care mapping**.

**Figure 3:**
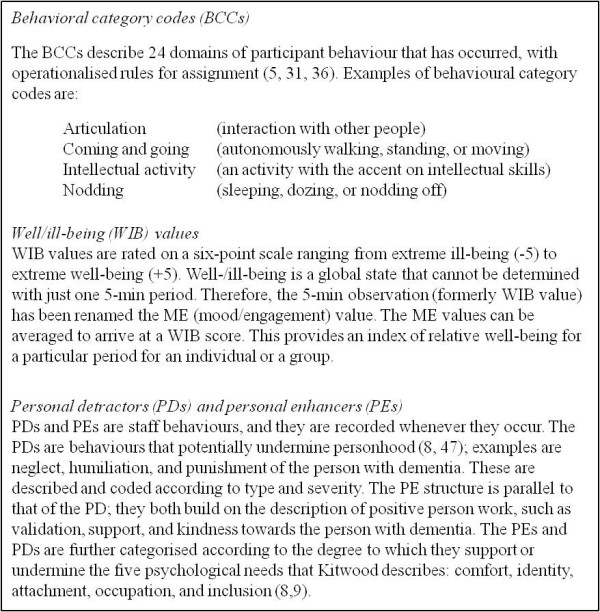
**Explanation of BCC, WIB, PD's en PE's**.

After completing the DCM training and the DCM organisational briefing day, the intervention nursing homes will carry out two DCM cycles. A single DCM cycle (Figure 2) consists of:

1. *Observation*. An observer (mapper) continuously observes an average of five (four to six) residents with dementia for a representative period (a minimum of 4 h/day) in communal areas (living rooms or common rooms) of care facilities. After each 5-min period (a time frame) a coding protocol will be used to record what has happened to each participant and what the behaviour of the staff was [20,30]. Dementia-care mapping employs behavioural category codes (BCCs), well/ill-being (WIB) values, personal detractions (PDs), and personal enhancers (PEs) to code this behaviour (Figure 3).

2. *Feedback*. The results of the observation are fed back to the staff. The positive communication style of the feedback enables them to interpret it in the context of the residents' lives rather than relating it to themselves in a negative way. The feedback style enables the staff to form a more complete picture of the residents and prevents resistance to negative feedback or unwillingness to change their personal style of care.

3. *Action plans*. The staff draw up action plans for care improvements at an individual level and a group level on the basis of feedback discussions. Action plans are tools for implementing the principles of person-centred care in daily practice.

### Control group

Caregivers in the control group will receive neither the DCM training nor the DCM organisational briefing day. The control group residents will continue to receive usual care during the trial. To motivate these nursing homes to complete the measurements, a researcher will visit each control nursing home at the start of the trial, and the control nursing homes will receive the DCM training after the trial.

### Measurements

The study outcome variables will be measured at the resident and staff levels. The primary outcome measure is resident agitation, to be assessed with the Cohen-Mansfield Agitation Inventory (CMAI). This questionnaire consists of 29 items about agitation and aggression in residents with dementia, and it has been validated for use in the Netherlands [33,34]. The secondary outcome measures are the residents' other neuropsychiatric symptoms, to be assessed with the Neuropsychiatric Inventory - Nursing Homes (NPI-NH), a comprehensive neuropsychiatric rating scale including the following symptoms: delusions, hallucinations, agitation, depression, anxiety, euphoria, apathy, disinhibition, irritability, aberrant motor behaviour, night-time disturbances and eating change [35]. The residents' quality of life will be measured with Qualidem [36] and EQ-5D [37]. We will use the Global Deterioration Scale (GDS) to obtain information about dementia severity [38]. Such information will include fall incidents, physical restraints, and the amount of care delivered, which is recorded in the nursing-home administration system. A questionnaire about the resident demographics at baseline has been developed for our study, and it includes the following variables: age, sex, marital status, highest completed education, country of origin, longest former profession, and co-morbidity.

The following staff outcome measures will be collected: stress-related symptoms, job experience, job satisfaction, job-stress-related absenteeism, and employee turnover. We will use the General Health Questionnaire (GHQ-12) to measure stress-related symptoms. This validated instrument consists of 12 questions, and it is sensitive for measuring changes in general health [39,40]. We will also use two validated Dutch questionnaires: the Questionnaire about Experience and Assessment of Work (QEAW) and the Maastricht Job Satisfaction Scale for Healthcare (MJSS-HC) [41,42]. The questionnaire about staff demographics at baseline was developed for the present study and consists of the following variables: age, sex, marital status, highest completed education, country of origin, and experience with person-centred care.

All staff members of the participating units will be asked to fill in questionnaires about themselves (MJSS-HC, QEAW, and GHQ-12). Any staff member who is the caregiver primarily responsible for a particular resident will also be asked to fill in questionnaires about the resident (CMAI, NPI-NH, Qualidem, EQ-5D and GDS; Table 1). The staff will use an internet application with a personal user name and password to fill in these questionnaires. All the variables will be measured at baseline (T_0_), after the first DCM cycle (T_1_), and after the second DCM cycle (T_2_).

**Table 1:** Data sources for measurements of residents and staff

Residents		
**Variable**	**Instrument/source**	**Type of variable**

Demographic variables	Self-developed questionnaire	Control variables

Dementia severity	Global Deterioration Scale	Control variable

Care needs	Weight of Care Package: nursing home administration	Control variable

Agitation	Cohen-Mansfield Agitation Inventory	Primary outcome/ICER

Neuropsychiatric symptoms	Neuropsychiatric Inventory - Nursing Homes	Secondary outcome

Quality of life	Qualidem and EQ-5D	Secondary outcome/ICER

Fall incidents	Nursing home administration	Secondary outcome/ICER

Physical restraints	Nursing home administration	Secondary outcome/ICER

Amount of care delivered and medication use	Nursing home administration	Secondary outcome/ICER

**Staff**		

**Variable**	**Instrument/source**	**Type of variable**

Demographic variables	Self-developed questionnaire	Control variables

Stress-related symptoms	General Health Questionnaire-12	Secondary outcome/ICER

Job experience and job assessment	Questionnaire about Experience and Assessment of Work	Secondary outcome/ICER

Job satisfaction	Maastricht Job Satisfaction Scale for Health Care	Secondary outcome/ICER

Stress-related absenteeism	Nursing home administration	Secondary outcome/ICER

Employee turnover	Nursing home administration	Secondary outcome/ICER

Quantitative and qualitative methods will be used in process analyses. Quantitative process analyses will help account for the possible differences in intervention 'dosage' that might moderate the effects of the DCM. Qualitative process analyses will be used to determine relevant facilitators of and barriers to further implementation.

### Economic data

The cost-effectiveness of the intervention will be calculated and compared to usual practice. Table 1 shows the various data sources for the assessment of resource use, direct costs and staff productivity losses. We ask all organisations and residents (or their family or legal guardian) permission to extract data from the nursing-home administration system. Intervention costs, including costs for the DCM training, will be estimated. Study-specific costs, which would not occur in routine application, will not be considered.

### Sample size calculations

The calculation of the sample size calculation includes two steps:

1. Chenoweth et al. [43] report that the treatment-control difference was 10.9 in their recent cluster-randomised controlled trial, which had with five units in the control group and five in the DCM group, a 20% attrition rate in 8 months, and an average of 14 evaluable patients at follow-up. As the 95% confidence interval of the mean difference was 0.7 - 21.1, the standard error of the difference was approximately (21.1- 0.7)/4 = 5.1. Therefore, a study with a similar attrition rate, standard deviation, cluster (unit) sizes, interclass correlation coefficient (ICC), analysis method and design, but with nine clusters per arm, would have a standard error of difference of approximately 5.1√(5/9) = 3.8. For a true difference between the treatments of 10.9, the power of such a study would be 80% (two-sided testing at alpha = 0.05).

2. In our study, we plan to include at least five organisations in the control group and at least five organisations in the intervention group, with an average of three units in each organisation. Due to the correlation, the 'effective' sample size for each arm will be

$$\begin{array}{c} \frac{number\;of\;units\;per\;arm}{[1+\left(number\;of\;units\;per\;organisation-1\right)}\\ \times \left(correlation\;of\;units\;within\;organisation\right)] \end{array}$$

Allowing the correlation between units within a organisation to be 0.3 at most (which is a safe margin), we would need 15 units/arm to have an 'effective' sample size of 9 units/arm. Using step 1, we conclude that, with at least 15 units/arm, along with an attrition rate, standard deviation, cluster (or unit) size, and an ICC (for patients within a unit) similar to those of Chenoweth et al. [43], we would have 80% power to detect a true difference of 10.9 between the treatment group and the control group.

### Statistical analyses

The effects on the primary outcome will be evaluated by means of linear mixed-effect models with treatment, baseline measures, and control variables (used in the sequential balancing minimisation procedure [25]) as covariates and the DSCU as a random effect, to correct for dependencies within DSCUs. We use intention to treat analysis and subgroup analysis were we compare the observed patients with the control group. We will use structural equation modelling in the secondary analysis to evaluate the plausibility of a theoretical model including a number of mediator variables (WIB and PE/PD). We will use quantitative methods to study the effectiveness, efficiency, and factors that can influence the implementation of DCM in the organisation. We intend to evaluate focus groups and determine relevant facilitators of and barriers to implementation by means of qualitative methods.

### Economic evaluation

The cost-effectiveness analyses focus on the addition of the DCM intervention to nursing homes and comparing it to usual care from a societal perspective. On the basis of the above-mentioned outcomes, two different incremental cost-effectiveness ratios (ICERs) will be computed: costs per quality-adjusted life year gained (by residents) and costs per increase in scores on staff job satisfaction measure (MJSS-HC). Other outcome measures such as neuropsychiatric symptoms and volumes of care, work stress, stress-related absenteeism and staff turnover will be financially valued and included in the ICER on the cost side. Cost-effectiveness will be analysed in a Bayesian fashion, i.e. we will derive an acceptability curve that can evaluate efficiency in a set of increasing thresholds for the denominators of the ICERs. Furthermore, cost-effectiveness analysis will be accompanied by the value of the information analysis.

## Discussion

A strength of DCM is that it offers an integral person-centred approach to dementia care in nursing-home settings. In addition to psychosocial interventions (action plans) focusing on individual staff members and residents, DCM also induces systematic adaptations in management style and organisation climate. We can expect that all these conditions need to operate synergistically if we are to sustainably improve effectiveness, efficiency, and quality of dementia care in nursing homes.

The major strengths of the study design are the large sample size, cluster randomisation, and a follow-up of 1 year. We will randomise clusters after recruiting the study sample and seeking informed consent from the residents. In this way, we can control for potential selection bias in the control and intervention groups. We will use the minimisation method for randomisation to assure an equal distribution of baseline characteristics. However, it is possible that both the intervention and the control nursing homes in our study are more than averagely motivated to implement person-centred care. Any implementation strategies developed on the basis of our findings may therefore have suboptimal generalisability. However, in this respect, no differences are to be expected between the intervention and the control groups. The effect of the DCM intervention could perhaps be underestimated because nursing home organisations in the control group may already have a more positive attitude towards person-centred care than the average nursing-home organisation in the Netherlands. We will collect data from previous person-centred-care track records for all nursing homes in the study.

In this study, we will first train the staff from the intervention nursing homes before taking baseline measurements. The purpose of this is to minimise the attrition rate; the period from the start of the training and the end of the first DCM cycle is 9 months. Due to the decision to train the staff before the baseline measurement, it is conceivable that training might affect the behaviour of the trained staff member in that he or she may already start applying the principles of person-centred care in daily practice. Obviously, this could influence care giving in the intervention nursing homes before the baseline measurement. In order to attenuate contamination, the staff will be instructed not to disclose or try to implement the DCM method or person-centred care until the organisational briefing day has taken place. Possible baseline differences will be accounted for by their inclusion in the analyses.

From a public health perspective, this study should provide evidence regarding the effectiveness of nonpharmacological support for dementia patients in nursing homes in the Netherlands. It is necessary for policymakers to make their decisions about financing new services on the basis of strong evidence regarding the acceptance of new interventions and their cost-effectiveness.

## Competing interests

This study was funded by the Netherlands Organisation for Health Research and Development (ZonMw). GvdV and ID were financially supported by the funding bodies. The funding bodies did not play a role in any part of the study. The other authors declare that they have no competing interests.

## Authors' contributions

ID was responsible for the research proposal. ID, AP, and GvdV designed the study. GvdV wrote the first draft of the manuscript and was responsible for revisions. ID and MV contributed to the drafting of the manuscript. RG and EA gave advice on the statistical analysis and the economic analysis, respectively. SZ and RK commented on the design and the manuscript. All authors have read and approved the final manuscript.

## Pre-publication history

The pre-publication history for this paper can be accessed here:

http://www.biomedcentral.com/1471-2318/12/1/prepub
